# Genome-wide identification and comprehensive analysis reveal potential roles of long non-coding RNAs in fruit development of southern highbush blueberry (*Vaccinium corymbosum* L.)

**DOI:** 10.3389/fpls.2022.1078085

**Published:** 2022-12-13

**Authors:** Shuigen Li, Jiaying Zhang, Liqing Zhang, Xianping Fang, Jun Luo, Haishan An, Xueying Zhang

**Affiliations:** ^1^ Forest and Fruit Tree Research Institute, Shanghai Academy of Agricultural Sciences, Shanghai, China; ^2^ Shanghai Key Lab of Protected Horticultural Technology, Shanghai Academy of Agricultural Sciences, Shanghai, China

**Keywords:** blueberry, fruit development, lncRNA, RNA-seq, anthocyanin biosynthesis, flavonoid biosynthesis

## Abstract

**Introduction:**

Blueberries have a high antioxidant content and are produced as healthy food worldwide. Long non-coding RNAs (lncRNAs) are a type of regulatory RNAs that play a variety of roles in plants. Nonetheless, information on lncRNAs and their functions during blueberry fruit development is scarce in public databases.

**Methods:**

In the present study, we performed genome-wide identification of lncRNAs in a southern highbush blueberry using strand-specific RNA sequencing (ssRNA-Seq). Differentially expressed lncRNAs (DE-lncRNAs) and their potential target genes were analyzed at four stages of fruit development. Cis-regulatory DE-lncRNAs were predicted using co-localization analysis.

**Results:**

These findings included a total of 25,036 lncRNAs from 17,801 loci. Blueberry lncRNAs had shorter transcript lengths, smaller open reading frame (ORF) sizes, fewer exons, and fewer isoforms than protein-coding RNAs, as well as lower expression levels and higher stage-specificity during fruit development. A total of 105 DE-lncRNAs were identified among the comparison group of PAD vs. CUP, 443 DE-lncRNAs were detected when comparing CUP with PINK fruits, and 285 DE-lncRNAs were revealed when comparing PINK and BLUE fruits. According to Kyoto Encyclopedia of Genes and Genomes annotation, target genes of DE-lncRNAs were primarily enriched in the “Autophagy-other”, “DNA replication”, “Endocytosis”, ‘photosynthesis’ and ‘chlorophyll metabolism’ pathways, suggesting that lncRNAs may pay potential roles in fruit expansion and ripening. Moreover, several lncRNAs have been proposed as cis-regulators of the key genes involved in flavonoid biosynthesis. *MSTRG.107242.6*, and its putative target gene, BTB/POZ and TAZ domain-containing protein, might play critical roles in anthocyanin accumulation in blueberries.

**Discussion:**

These findings highlight the regulatory function of lncRNAs and aid in elucidating the molecular mechanism underlying blueberry fruit growth.

## Introduction

Blueberry (*Vaccinium corymbosum*, Ericaceae) is a perennial shrub that has become a high-value fruit crop worldwide. The blueberry fruits have a pleasant flavor and are a top source of antioxidants (Cao et al., 1998) that have been found to generate diverse positive impacts on human health (Neto, 2007; Basu et al., 2010; Shi et al., 2017). Understanding the molecular pathways underlying fruit development would help cultivars produce blueberries of higher fruit quality. Previous studies (Zifkin et al., 2011; Plunkett et al., 2018; Hou et al., 2020; Li et al., 2020b; Xin et al., 2021) have shown that specific transcription factors (TFs), plant hormones, and microRNAs are important in controlling blueberry fruit growth and color change. Although numerous intricate events occur during the biological process of berry fruit formation, the regulatory networks that control them remain poorly understood.

The onset of fruit development occurs at the end of double fertilization. According to principal component analysis, the blueberry fruit then undergoes three developmental stages: petal fall, fruit expansion, and maturation stages (Colle et al., 2019). The fundamental characteristic of the fruit expansion stage is a change in fruit size. According to a transcriptome study (Yang et al., 2021), the plant hormone signal transduction pathway is enhanced during the blueberry fruit growth. Jasmonate-related *TIFs* genes were also considered as potential elements affecting fruit size by controlling other phytohormones (Yang et al., 2021). Fruit maturation is characterized by color changes, texture softening, and nutrient accumulation. One of the main contributors to color change is polyphenolic anthocyanin pigment, which can have a significant impact on the quality of blueberry fruits. Researchers have been more interested in studies interpreting ripening-related regulators in blueberries. Abscisic acid may act as a growth regulator, causing ripening and regulating blueberry flavonoid biosynthesis (Zifkin et al., 2011). R2R3-MYB TFs function in the activation of anthocyanin biosynthesis in several species. In addition, the MYBPA1 is a transcriptional regulator of proanthocyanidin synthesis during the early developmental stage of blueberry fruits (Zifkin et al., 2011). MYBA transactivates the promoter of *DFR*, a crucial gene in the synthesis of anthocyanins, from blueberry and other species (Plunkett et al., 2018). Blueberry microRNAs were discovered using high-throughput short RNA sequencing, and it was hypothesized that these microRNAs would be crucial for fruit maturation and coloring by inhibiting the expression of auxin-responsive genes or those involved in fruit growth (Hou et al., 2017). Chlorophyll degradation is considered as another major factor that contributes to color change during blueberry fruit ripening. Anthocyanin biosynthesis and chlorophyll degradation in tomatoes were enhanced by a blueberry VcMIR156a, which was identified to posttranscriptionally regulate its target *VcSPL12* (Li et al., 2020b).

Transcriptome studies in eukaryotes have shown that pervasive transcription from more than 90% of the genome generates a larger number of non-coding RNAs (Chekanova et al., 2007; Kapranov et al., 2007; Chekanova, 2015). Non-coding RNAs have become star molecules and have attracted increasing attention from researchers (Urquiaga et al., 2021). A large number of non-coding RNAs have been sequenced and identified using high-throughput sequencing technologies. Functional analyses have shown that they play important roles in eukaryotes (Chekanova, 2015; Quinn and Chang, 2016; Wang and Chekanova, 2017) by interacting with protein-coding genes. These non-coding RNAs play important regulatory roles in plant biological processes such as organ development, immunity, and responses to environmental stimuli (Quinn and Chang, 2016). Regulatory non-coding RNAs include microRNAs, siRNAs, and long non-coding RNAs (lncRNAs). LncRNAs have more than 200 nucleotides (nt) and do not encode proteins but can generate small peptides (Ben Amor et al., 2009). Plant genomes harbor a large number and diverse population of lncRNAs, but much less is known about their presence and function than those of small non-coding RNAs (Ben Amor et al., 2009). The regulatory function of lncRNAs is achieved by their interaction with proteins or other non-coding RNAs (Wang and Chekanova, 2019). They can regulate gene expression by affecting promoters, untranslated regions, exons, introns, and terminators (Wierzbicki et al., 2021). However, it is a major challenge to deduce the function of lncRNAs because they lack a protein-coding domain and are often not conserved. Most lncRNAs are functionally or mechanistically connected to mRNA expression (Wierzbicki et al., 2021), and thus we can infer the function of lncRNAs by analyzing co-expression. If an lncRNA exhibits a high degree of correlated expression with a protein-coding gene across diverse developmental stages, it may imply that they have some functional connections (Kang and Liu, 2019) and the protein-coding gene is considered to be a *trans*-target. Because some lncRNAs may also function in *cis* to regulate the transcription of neighboring protein-coding genes (Ariel et al., 2014), *cis*-target genes can be proposed depending on the relative genomic locations of lncRNAs and protein-coding genes.

LncRNAs have also been shown to be important regulators of fruit development. *LncRNA1459* is a positive element during tomato fruit ripening that affects ethylene production and lycopene accumulation, and loss-of-function mutants of *LncRNA1459* significantly repress the tomato ripening process (Li et al., 2018). *MdLNC499* is an apple lncRNA that functions in light-induced fruit coloration by inducing *MdERF109* expression (Ma et al., 2021). Using next-generation sequencing techniques, a large number of lncRNAs that participate in the fruit developmental process have been identified in several fruit species, including strawberry (Kang and Liu, 2015), tomato (Zhu et al., 2015; Wang et al., 2018), kiwifruit (Tang et al., 2016; Chen et al., 2021), sea buckthorn (Zhang et al., 2017), *Cucumis melo* (Tian et al., 2019), and apple (Yang et al., 2019). However, the presence of lncRNAs in blueberries has not been studied. In contrast to protein-coding mRNAs, many lncRNAs exhibit lower levels of sequence conservation (Dinger et al., 2008; Wierzbicki et al., 2021) and show organ-, development-, and environment-specific expression (Zhang et al., 2013). Therefore, the identification of fruit-related lncRNAs is required to improve our understanding of blueberry fruit development.

Benefitting from the completion of tetraploid blueberry genome sequencing (Colle et al., 2019), the present study performed genome-wide identification and analysis of lncRNAs. We also analyzed differentially expressed lncRNAs (DE-lncRNAs) and proposed their potential function during fruit expansion and ripening. These results will pave the way for interpreting the molecular mechanisms of lncRNA regulation underlying blueberry fruit development.

## Materials and methods

### Plant materials and growth conditions

**Figure 1:**
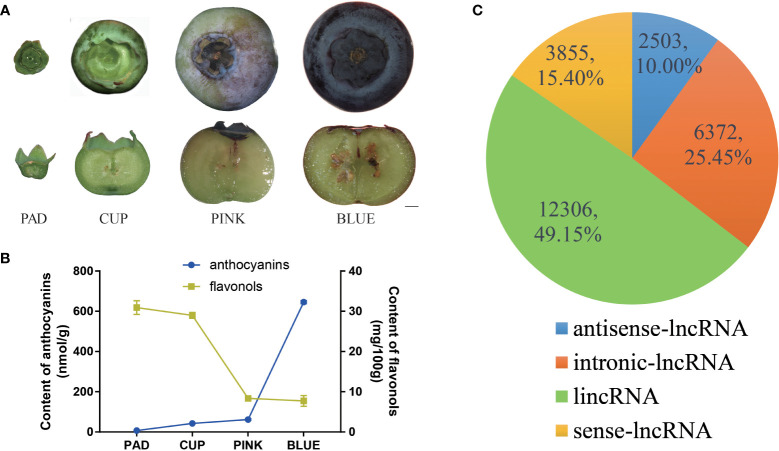
Identification and classification of blueberry lncRNAs in blueberry. **(A)** Whole phenotype and longitudinal section of ‘Misty’ fruits at the four developmental stages. Scale bar = 2 mm. **(B)** The content of anthocyanin and flavonols. **(C)** All lncRNAs were classified as long intergenic non-coding RNAs (lincRNAs), antisense lncRNA, intronic lncRNA, and sense lncRNA, with the number and percentages shown in the pie chart.

Three-year-old trees (*V. corymbosum* ‘Misty’) were cloned using cuttage and grown under greenhouse conditions at the Shanghai Academy of Agricultural Sciences (Shanghai, China). Approximately 200–300 fruits were randomly picked from five different plants and sorted into four developmental stages: pad fruits, cup fruits, pink fruits, and blue fruits (**Figure 1A**). All fruits were frozen immediately in liquid nitrogen and stored at −80°C.

### Extraction and content determination of anthocyanins

Anthocyanins were extracted and assayed according to the method of Pirie (Pirie and Mullins, 1976) with minor modifications. The fruits at the four developmental stages were fully ground into powder in liquid nitrogen. Aliquots of 1.0 g ground fruit tissue were dissolved initially in 5 ml of an aqueous methanolic HCl solvent (methanol, 0.1% concentrated HCl, approximate pH of 3.5) and then kept at 4°C and dark condition for 24 h. After centrifugation at 12,000 rpm for 10 min, the supernatant was transferred to a 10-ml volumetric flask and diluted to a final volume of 10 ml with methanolic HCl solvent.

Total anthocyanins were assayed by measuring absorbances at 530 nm using a microplate reader. To eliminate the interference of chlorophyll, the absorbance was measured at 620 and 650 nm. The optical density of anthocyanins was calculated using the following formula:


$$OD\lambda =A_{530}-A_{620}-0.1\times \left(A_{650}-A_{620}\right)$$

where A530, A620, and A650 are the absorbance values at 530, 620, and 650 nm, respectively. Anthocyanin content was calculated using the following formula:


$$C\left(\text{nmol}/\text{g}\right)=OD\lambda /\xi \lambda \times V/m\times 10^{6}$$

where ξλ is 4.62 × 10^4^ for molecular extinction coefficient of anthocyanin at 530 nm, V is 10 ml of the final volume, and m is 1.0 g of the sample weight.

### Measurement of total flavonols

The content of flavonols was determined according to a well-established method (Miliauskas et al., 2004) using rutin standards. One gram of fruit sample was ground into powder and then ultrasonically extracted for 30 min. After centrifugation at 8,000 rpm for 10 min, the supernatant was used for subsequent measurements. All determinations were carried out in three replicates.

### RNA extraction, cDNA library construction, and RNA sequencing

Total RNA was extracted from the fruits of four separate pool samples using the RNAprep Pure Plant Kit (Tiangen, Beijing, China) according to the manufacturer’s protocol. Three biological replicates per sample were combined from 10 fruits each. Genomic DNA was removed from total RNA using DNase I treatment. RNA degradation and contamination were monitored by 1% agarose gel electrophoresis. A Qubit^®^ RNA Assay Kit with a Qubit^®^ 2.0 Fluorometer (Life Technologies, CA, USA) was used to measure RNA concentration. The RNA Nano 6000 Assay Kit for the Agilent Bioanalyzer 2100 system (Agilent Technologies, CA, USA) was used to assess RNA integrity.

First, ribosomal RNA (rRNA) was depleted from the total RNA using the Ribo-Zero™ Magnetic Kit (Epicenter, WI, USA) according to the manufacturer’s recommendations. The rRNA-depleted mRNA was then fragmented into small pieces. Finally, four highly strand-specific cDNA libraries were generated using an improved method (Parkhomchuk et al., 2009). The constructed libraries were submitted to Biomarker Technologies (Beijing, China) for 150-bp paired-end sequencing on the NovaSeq 6000 Sequencing System at a depth of ~67 million reads per library. All sequencing data were submitted to NCBI under the Bioproject accession PRJNA846130.

### Transcript assembly

Raw data were processed by removing reads containing adapters, reads containing ploy-N, and low-quality reads. At the same time, the Q20, Q30, and GC (guanine and cytosine) content of the clean data were calculated. HISAT2 v2.0.4 (Kim et al., 2015) was used to map the clean data to the reference genome (http://gigadb.org/dataset/100537) (Colle et al., 2019). The transcriptome was assembled using the StringTie v1.3.1 (Franceschini et al., 2012) based on clean data mapped to the reference genome.

### Identification of long non-coding RNA by bioinformatic analysis

The assembled transcripts were annotated by comparison with the blueberry genome-annotated protein sequences using the gffcompare program (Cuffcompare v2.1.1, http://cole-trapnell-lab.github.io/cufflinks/manual/). Unannotated transcripts were used to screen putative lncRNAs. In addition, transcripts longer than 200 nt and more than two exons were selected as lncRNA candidates. Transcripts with FPKM (fragments per kilobase million) <0.1 were discarded. Next, Coding Potential Calculator (CPC2) (Kong et al., 2007), Coding-Non-Coding Index (CNCI) (Sun et al., 2013), and Coding Potential Assessment Tool (CPAT) (Wang et al., 2013) were combined to test the protein-coding potential. Only transcripts that did not pass any of the above three tests were considered non-protein-coding RNA candidates. The resulting transcripts were aligned to the Pfam protein database (Finn et al., 2014) to filter the transcripts containing a known protein domain. Finally, to detect microRNA precursors, putative lncRNAs were blasted against blueberry microRNA precursors from PmiREN 2.0 (https://www.pmiren.com/). LncRNAs with an E-value <1e-5 were potential microRNA precursors and were excluded. Different types of lncRNAs including long intergenic non-coding RNAs (lincRNAs), intronic lncRNAs, antisense lncRNAs, and sense lncRNAs were selected using Cuffcompare v2.1.1.

### Predicting *cis-*/*trans-*target genes of long non-coding RNA

The *cis-* and *trans*-target genes of lncRNAs were predicted based on the interaction between lncRNA and its target genes. Some lncRNAs may function in *cis* to regulate the transcription of neighboring protein-coding genes. In this study, we firstly identified lncRNAs and their neighboring genes. Both the upstream and downstream neighboring genes of all lncRNAs at a distance of 100 kb were identified using Perl scripts. The *trans*-target genes of lncRNAs were predicted by correlation analysis of lncRNA and mRNA expression among the samples. The Pearson correlation coefficient (PCC) method was used to analyze the correlation between the lncRNAs and mRNAs. Genes with absolute correlation values >0.9 and significant P-values <0.01 were selected as the *trans*-target genes of lncRNA.

### Differential expression analysis and construction of the co-localization networks

StringTie v1.3.1 (Franceschini et al., 2012) was used to calculate FPKMs of both lncRNAs and protein-coding genes in each sample. DE-lncRNAs and differentially expressed mRNAs (DEGs) between the two groups were determined using the DESeq R package (1.10.1) (Anders and Huber, 2010) based on the negative binomial distribution. The resulting P-values were adjusted using the Benjamini–Hochberg approach (Benjamini and Hochberg, 1995) for controlling the false discovery rate (FDR). LncRNAs were considered differentially expressed when showing fold change ≥2 and FDR <0.01.

To construct co-localization networks of DE-lncRNAs and DEGs, PCCs were calculated using the FPKMs of the lncRNAs *vs*. mRNAs of their neighboring upstream and downstream genes. Only the lncRNA and mRNA with a genomic distance <10 kb and absolute PCC ≥0.7 were considered *cis*-regulatory lncRNAs that formed a co-localization pair. The interactions of the co-localization pairs were analyzed using Cytoscape 3.9.1 (Shannon et al., 2003).

### Functional annotation of differentially expressed long non-coding RNA targets

Both *cis*- and *trans*-targets of the DE-lncRNAs were used for functional annotation. Gene Ontology (GO) enrichment analysis was performed using TopGo (R package). KOBAS (Mao et al., 2005) software was used to test the statistical enrichment of DE-lncRNA targets in the Kyoto Encyclopedia of Genes and Genomes (KEGG) pathways.

### Quantitative reverse transcription-PCR

To verify the results of RNA sequencing (RNA-seq) analysis, we randomly selected six DE-lncRNAs and three DEGs. Gene-specific primers for each transcript were designed using Primer 5 software (Lalitha, 2000), and all primer sequences are listed in **Supplementary Table S1**.

The expression levels of the selected lncRNAs were detected in four sets of fruits, with three replicates for each sample. Total RNA was extracted using the CTAB method (RNAprep Pure Plant Kit, Tiangen, China). Next, 1 mg of total RNA, treated with RNase-free DNase to eliminate genomic DNA, was reverse transcribed into cDNA using PrimeScript™ RT reagent Kit with gDNA Eraser (Takara, Japan). After dilution, 2 μl of the cDNA solution was subjected to real-time PCR amplification using the SYBR Premix EX Taq Kit (Takara, Japan). Then, the qRT-PCR reactions were performed on the LC480 System (Roche, USA). After PCR, dissociation curves were generated to verify the amplification specificity. The data are the averages of three replicates and were calculated using the relative quantitative analysis method. All expression levels were normalized to that of the internal control, *glyceraldehyde-3-phosphate dehydrogenase (GAPDH)*.

## Results

### Identification of long non-coding RNAs from RNA sequencing databases of blueberry fruits

The southern highbush blueberry ‘Misty’ was selected for this study. ‘Misty’ produced large fruits with a high yield per plant and was demonstrated strong growth adaptability in Shanghai, China. To investigate fruit development-related lncRNAs in blueberries, the developmental process of ‘Misty’ fruits was first observed, and four distinct stages of fruits were harvested (**Figure 1A**). The fruits grew from pad fruits, which had green skin and hard flesh, as well as small and clear zygotic embryos. The fruits then continued to develop into cups that grew in size, and the skin color changed to light green. Fruits began to change color from the PINK stage and developed a hard texture, with parts of the skin and flesh turning pink. Fruit and embryos rapidly expanded from CUP to PINK fruits. During the maturation phase, the size of the fruit changed insignificantly from PINK to BLUE, whereas an obvious color change occurred. The maturation process of the fruits was completed at the BLUE stage, which showed a softened texture with a dark blue skin and white flesh. Anthocyanin content was gradually increased during the fruit developmental process. The pad fruits contained extremely low content of anthocyanins. Cup and pink fruits gradually accumulated more anthocyanins, but the content was still at a low level. Approximately 10 times more anthocyanins were found in blue fruits than in pink fruits (**Figure 1B**). In contrast, the flavonol content revealed a decreasing trend during the fruit developmental process (**Figure 1B**).

The RNA-seq libraries were constructed using ‘Misty’ fruits picked at four distinct developmental stages with three biological replicates each (**Figure 1A**). After ribosomal RNA (rRNA) was depleted from the total RNA, strand specific RNA sequencing (ssRNA-seq) was performed on 12 blueberry fruit libraries. A total of 125.39 Gb clean data and 9.56 Gb per sample were obtained after filtering out adaptor sequences and reads of low quality (**Supplementary Table S2**). The Q20 and Q30 values of the clean data exceeded 98.11% and 94.78%, respectively. The percentage of reads mapped to the blueberry reference genome in each library ranged from 69.44% to 82.91%. More than 56.2% of reads were mapped to exons, and approximately 15.5%–25.8% of the reads were mapped to the intergenic region. Approximately 12.8%–18.0% of the reads per sample were mapped to introns.

The transcriptome was assembled using reads that mapped to the reference genome. After analysis of the protein-coding potential with CPC2, CNCI, CPAT and Pfam scan, 25,150 non-protein-coding RNAs were identified from all transcripts. Subsequently, 114 transcripts were predicted to be microRNA precursors. Finally, a total of 25,036 lncRNAs from 17,801 loci were identified. Among these lncRNAs, 12,306 were classified as lincRNAs, 2,503 as antisense lncRNAs, 6,372 as intronic lncRNAs, and 3,855 as sense lncRNAs (**Figure 1C**).

### Characterization of blueberry long non-coding RNAs

**Figure 2:**
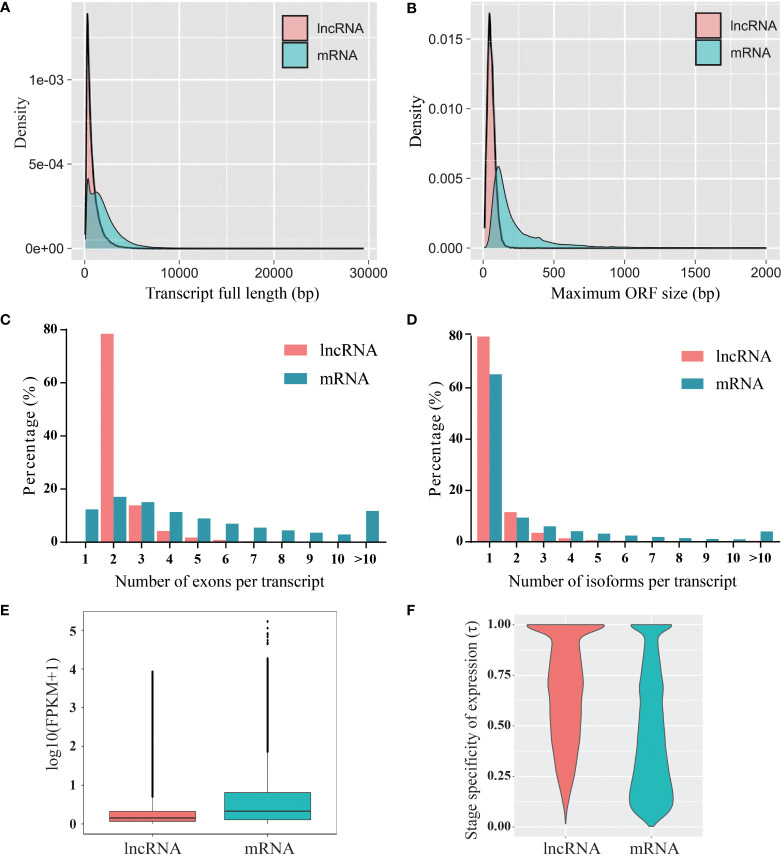
Characterization of blueberry lncRNAs compared to that of mRNAs. **(A)** Transcript length, **(B)** open reading frame (ORF) length, **(C)** exon number, **(D)** isoform number, **(E)** expression levels, and **(F)** expression specificity.

The characterization of lncRNAs was performed by comparison with protein-coding RNAs. Compared to the protein-coding RNAs, the length of the lncRNAs was shorter, and most of the lncRNAs (72%) were shorter than 1,000 nt (**Figure 2A**). In addition, the maximum ORF size of the lncRNAs was shorter than that of the protein-coding RNAs (**Figure 2B**). The transcripts of protein-coding RNAs contained a greater number of exons than that of lncRNAs, and most of the lncRNAs (78.5%) contained two exons (**Figure 2C**). The distribution of lncRNA isoforms was similar to that of protein-coding RNAs; however, lncRNAs contained fewer isoforms than protein-coding RNAs (**Figure 2D**). A comparison of the FPKM of whole transcripts indicated that the expression levels of lncRNAs were generally lower than those of protein-coding RNAs (**Figure 2E**). Furthermore, the expression specificity of lncRNAs and protein-coding RNAs was calculated using index τ method (Yanai et al., 2005). The expression pattern of lncRNAs during fruit development showed higher stage specificity than that of protein-coding RNAs (**Figure 2F**).

### Differential expression of mRNAs and long non-coding RNAs

**Figure 3:**
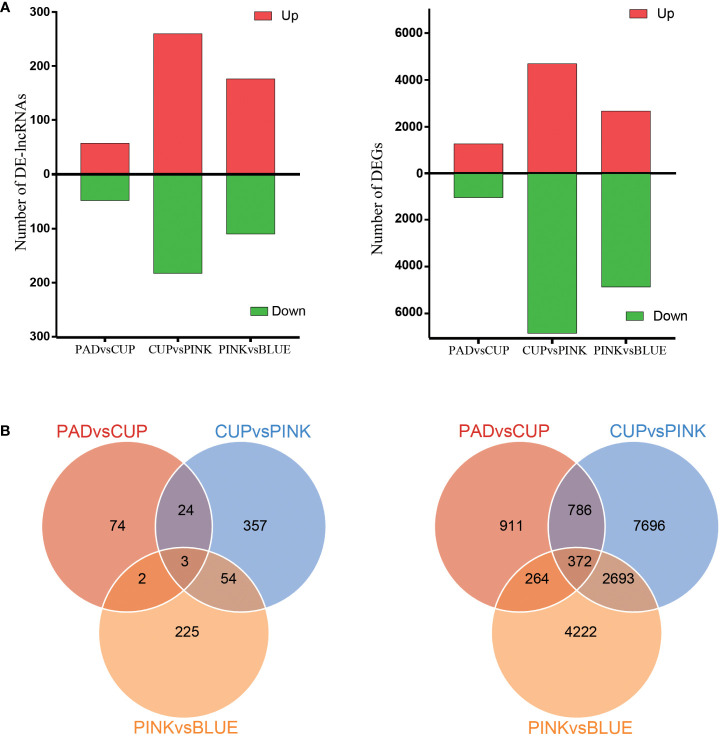
Analysis of differentially expressed lncRNAs and mRNAs. **(A)** Numbers of upregulated and downregulated lncRNAs (left) and mRNAs (right). **(B)** Venn diagrams of the numbers of differentially expressed lncRNAs (left) and mRNAs (right) between three comparisons.

The DEGs and DE-lncRNAs were selected based on an expression variation ratio of fold change ≥2 and significance of FDR <0.01. As a result, 2,333, 11,547, and 7,551 DEGs were identified in the PAD *vs*. CUP, CUP *vs*. PINK, and PINK *vs*. BLUE groups, respectively (**Figure 3A**). A comparison of the lncRNAs among the four samples identified 105 DE-lncRNAs in the PAD *vs*. CUP group, 443 in the CUP *vs*. PINK group, and 285 in the PINK *vs*. BLUE group (**Figure 3A**). There were 372 mRNAs and only three lncRNAs that were differentially expressed among all samples according to the Venn statistical analysis (**Figure 3B**). Maximum numbers of DEGs and DE-lncRNAs were both found at the developmental stages of CUP to PINK fruits, followed by the stages of PINK to BLUE fruits.

**Figure 4:**
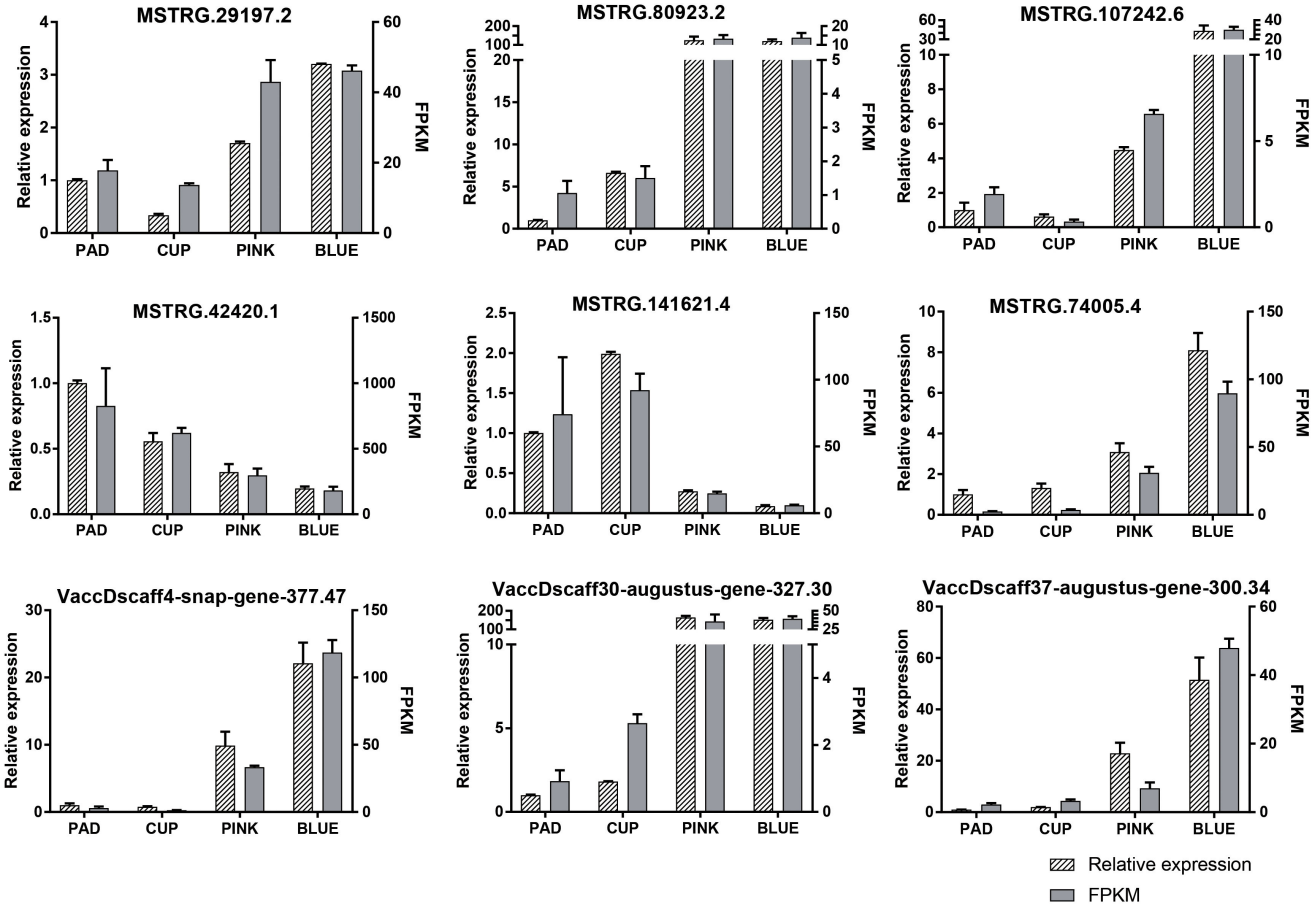
Expression patterns of nine randomly selected transcripts validated by qRT-PCR. The relative expression and fragments per kilobase million (FPKM) are both showed.

DE-lncRNAs and DEGs were verified by qRT-PCR. The expression of nine transcripts was highly coordinated with their PFKM (**Figure 4**), validating our transcriptome profiling analysis.

### Prediction and function annotation of differentially expressed long non-coding RNA targets

Potential *cis*- and *trans*-targets of DE-lncRNAs were predicted based on their genomic location and expression, respectively. GO analysis revealed that most of the differentially expressed target genes were enriched for “metabolic process” in the category of biological process, “cell” in cellular component, and “binding” in molecular function.

**Figure 5:**
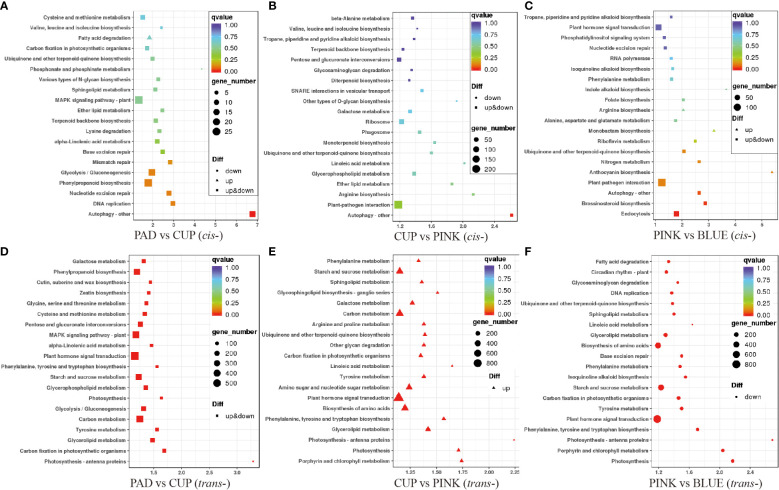
Enrichment analysis of target genes of differentially expressed lncRNAs (DE-lncRNAs) in Kyoto Encyclopedia of Genes and Genomes (KEGG) pathways. The ordinate represents the pathway name, and the abscissa represents the enrichment factor. Each dot in the figure represented a pathway, and the size indicates the number of genes enriched. The color of the dot represents P-value. Upregulated and downregulated genes are distinguished by triangle and circles, respectively. **(A–C)** show the cis-target genes of DE-lncRNAs among PAD vs. CUP, CUP vs. PINK and PINK vs. BLUE comparisons, respectively. **(D–F)** display the trans-target genes of DE-lncRNAs among the above three comparisons, respectively.

The KEGG pathway was used to further annotate the potential function of the lncRNAs. First, we analyzed the *cis*-targets of DE-lncRNAs in four samples (**Figure 5**). As a result, during the early phases of fruit expansion (PAD *vs*. CUP), the *cis*-target genes were enriched in “Autophagy-other,” “DNA replication,” “Phenylpropanoid biosynthesis,” “Glycolysis/Gluconeogenesis,” and “Mismatch repair” as top pathways (**Figure 5A**). The “Autophagy-other,” “Plant-pathogen interaction,” “Arginine biosynthesis,” “Ether lipid metabolism,” and “Glycerophospholipid metabolism” were identified as top pathways during the later fruit expanding stage (CUP *vs*. PINK) (**Figure 5B**). During the fruit ripening stage (PINK *vs*. BLUE), the top KEGG pathways included “Endocytosis,” “Brassinosteroid biosynthesis,” “Autophagy-other,” “Plant-pathogen interaction,” and “Anthocyanin biosynthesis” (**Figure 5C**). According to these findings, “Autophagy-other” was the most important enriched pathway in each of the three comparisons, receiving the highest richness factor throughout the early developmental stages and the third highest richness factor at the maturity stage.

Enrichment analysis of *trans*-targets was also performed. The results showed that *trans*-targets of DE-lncRNA were enriched in “Photosynthesis-antenna proteins,” “Carbon fixation in photosynthetic organisms,” “Glycerolipid metabolism,” “Tyrosine metabolism,” and “Carbon metabolism” among PAD *vs*. CUP (**Figure 5D**). Then, the *trans*-target genes of upregulated lncRNAs were enriched in “Porphyrin and chlorophyll metabolism,” “Photosynthesis,” “Photosynthesis-antenna proteins,” “Glycerolipid metabolism,” and “Phenylalanine, tyrosine and tryptophan biosynthesis” under the comparison of CUP *vs*. PINK (**Figure 5E**), but no pathways were enriched for downregulated *trans*-target genes. The pathways of “Photosynthesis,” “Porphyrin and chlorophyll metabolism,” “Photosynthesis-antenna proteins,” “Phenylalanine, tyrosine and tryptophan biosynthesis,” and “Plant hormone signal transduction” were identified as the top five pathways during the PINK to BLUE stages (**Figure 5F**). The genes enriched in the photosynthesis pathways and photosynthesis-antenna proteins were upregulated in the fruit expansion stage and downregulated in the fruit ripening stage, which should be emphasized.

### Co-localization networks of differentially expressed long non-coding RNAs and their adjacent genes

**Figure 6:**
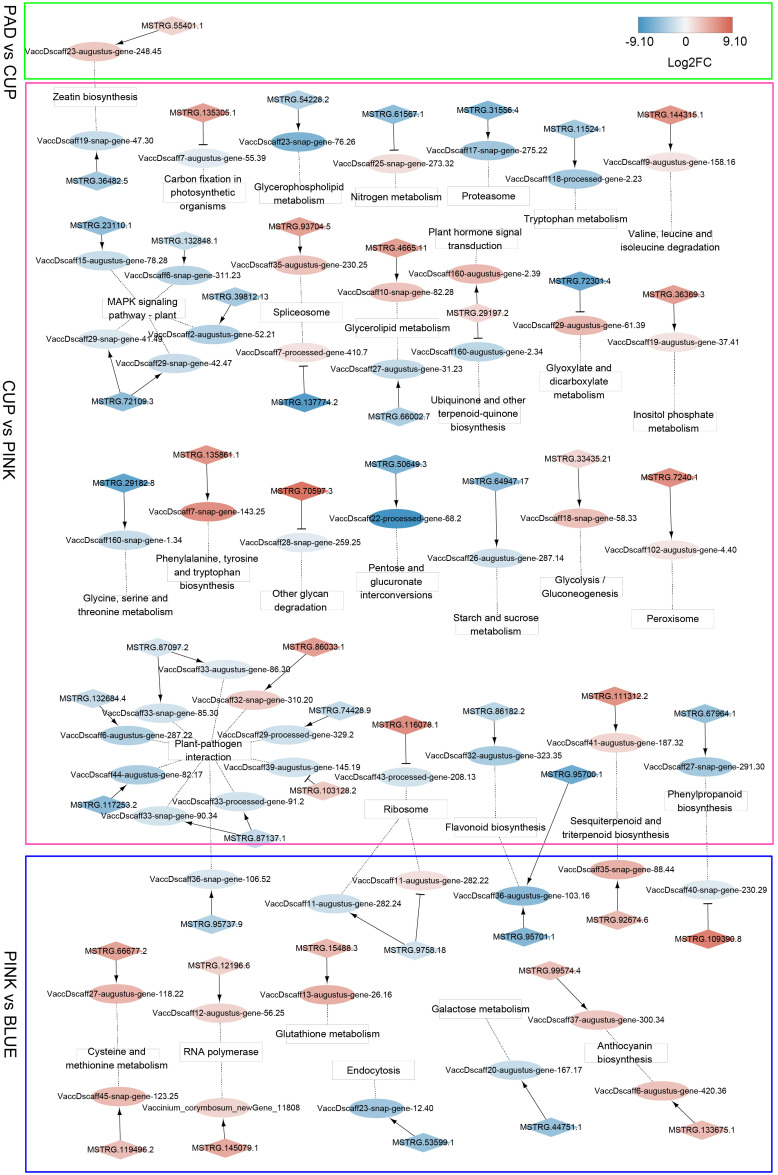
Co-localization networks of differentially expressed lncRNAs (DE-lncRNAs) and their adjacent genes enriched by Kyoto Encyclopedia of Genes and Genomes (KEGG) annotation. LncRNAs and protein-coding genes are indicated by diamonds and ellipses, respectively. KEGG terms are shown in square frames. The fold changes of transcripts are indicated by differential colors, with red and blue indicating upregulated and downregulated transcripts, respectively. Arrows indicate positive co-expression between lncRNAs and their target genes, and T-shaped lines indicate negative co-expression. Dotted lines connect the target genes and their KEGG terms.

Since some lncRNAs can function in *cis* to regulate the transcription of neighboring protein-coding genes, a co-localization analysis was performed to identify *cis*-regulatory lncRNAs and their adjacent genes. A total of 238 lncRNAs and 228 mRNAs were identified in the co-localization network (**Supplementary Table S3**). The interactions of these co-localized genes that were enriched by KEGG annotation are shown in **Figure 6**. In the PAD *vs*. CUP comparison group, we identified eight *cis*-regulatory DE-lncRNAs that formed eight regulatory pairs with eight DEGs. The gene (*VaccDscaff23-augustus-gene-248.4*5), which participates in zeatin biosynthesis, was positively regulated by an lncRNA (*MSTRG.55401.1*) and showed increased expression from PAD to CUP stages and decreased expression at the ripening stage. In the CUP *vs*. PINK group, 185 regulatory pairs (152 DE-lncRNAs and 155 DEGs) were identified. The largest number of *cis*-regulatory DEGs was enriched in “Plant-pathogen interaction” and “MAPK signaling pathway-plant” among the comparison of CUP *vs*. PINK. Additionally, two *cis*-regulatory DEGs were identified as key genes that function in flavonoid biosynthesis. During the ripening process (BLUE *vs*. PINK), we identified 118 co-localization pairs, containing 97 DE-lncRNAs and 82 DEGs. Two *cis*-regulatory lncRNAs were co-expressed with anthocyanin synthetase genes, which indicated that lncRNAs might participate in the regulation of anthocyanin biosynthesis in the blueberry fruit.

### Analysis of the expressions of the *cis*-regulatory long non-coding RNAs associated with anthocyanin accumulation

**Figure 7:**
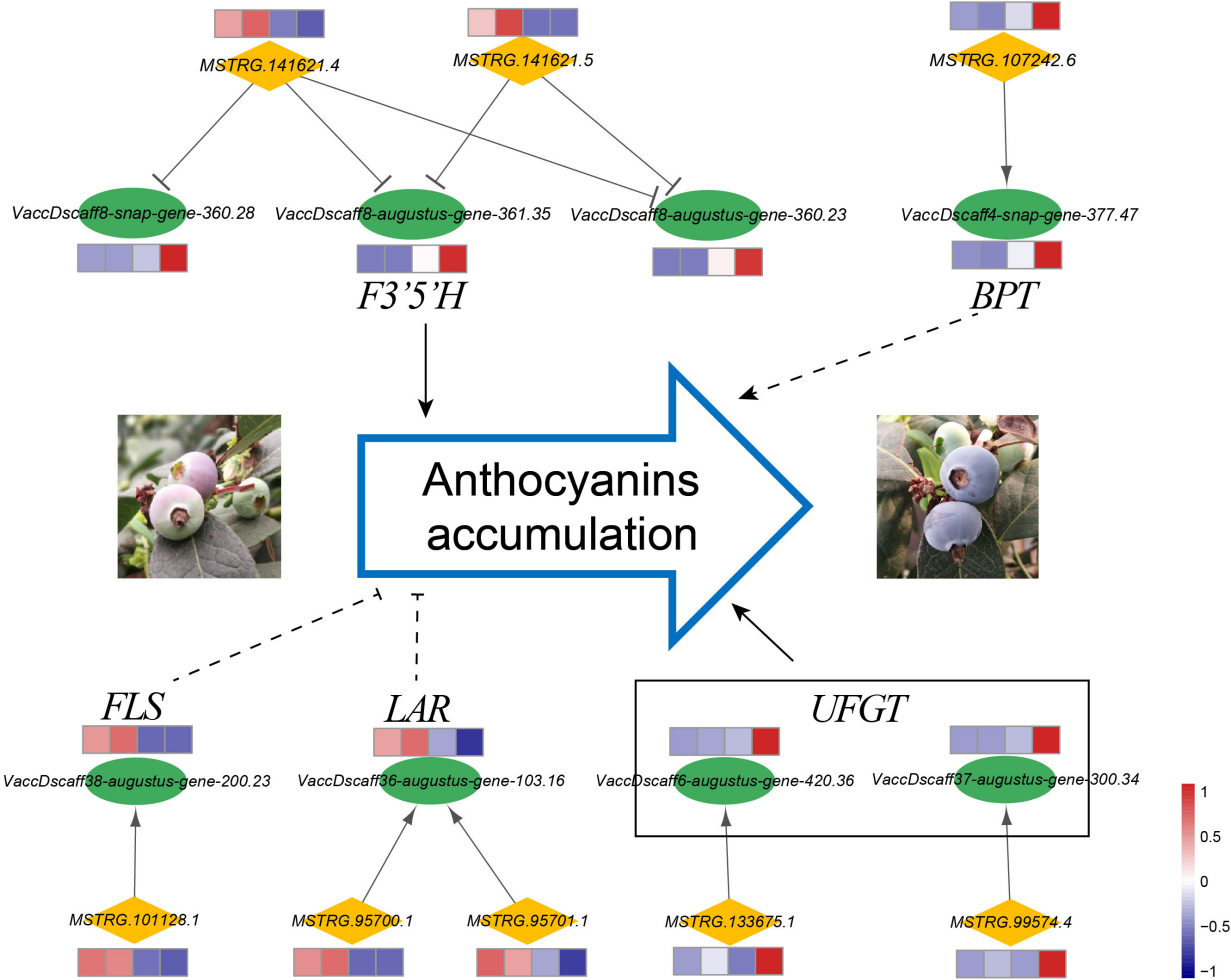
*Cis*-regulatory lncRNAs associated with anthocyanin accumulation. LncRNAs and protein-coding genes are indicated by orange diamonds and green ellipses, respectively. The expression patterns of transcripts are shown by heatmaps, and the expression levels at the four developmental stages are shown from left to right for each gene. The target genes were annotated as *flavonoid 3’,5’-hydroxylase* (*F3’5’H*), *BTB/POZ and TAZ domain-containing protein* (*BPT*), *flavonol synthase* (*FLS*), *leucoanthocyanidin reductase* (*LAR*), and *UDP-glucose: flavonoid 3-Oglucosyltransferase* (*UFGT*).

The flavonoid biosynthesis pathway in general leads to flavonols, anthocyanins, and proanthocyanidins (Zifkin et al., 2011). As anthocyanin is an important metabolite attributed to coloring and antioxidants, we analyzed the genes related to anthocyanin biosynthesis and proposed *cis*-regulatory lncRNAs that target these genes (**Figure 7**). Through bioinformatic analysis, several key genes that function in flavonoid biosynthesis pathways were inferred to be strongly co-expressed with their neighboring lncRNAs. The lncRNAs (*MSTRG.141621.4* and *MSTRG.141621.5*) derived from the same gene locus showed negative co-expression with three and two *F3’5’H* (*flavonoid 3’,5’-hydroxylase*) transcripts, respectively. The expressions of *F3’5’H* genes increased significantly during the fruit ripening stage, whereas the expressions of *MSTRG.141621.4* and *MSTRG.141621.5* showed decreasing trends. Association analysis revealed that the expressions of *F3’5’H* were coordinated with anthocyanin accumulation (correlation coefficient was >0.93). The *flavonol synthase* (*FLS*) gene and its potential *cis*-regulatory lncRNA (*MSTRG.101128.1*) had a positive correlation coefficient, and their expression levels decreased significantly at later developmental stages. The lncRNA *MSTRG.101128.1* and its target gene *FLS* were expressed in a highly coordinated manner with the accumulation of flavonols (the correlation coefficients were 0.999 and 0.983, respectively). We also observed that the *leucoanthocyanidin reductase* (*LAR*) gene showed much lower expression in ripening fruit than in early expansion fruits, and the *LAR* gene was inferred as the potential target of two lncRNAs, which were derived from two adjacent gene loci. Moreover, *MSTRG.99574.4* and *MSTRG.133675.1* could positively regulate their *cis*-target genes, which were annotated as two *UDP-glucose: flavonoid 3-O-glucosyltransferase (UFGT)* genes that participated in anthocyanin biosynthesis and showed higher expression at the ripening stage.

After Venn statistical analysis among the three comparison groups by co-localization analysis, only one *cis*-regulatory lncRNA (*MSTRG.107242.6*) was identified to be differentially expressed during the entire developmental process. The putative target gene of *MSTRS.107242.6* was the *VaccDscaff4-snap-gene-377.47*, which was annotated as a TF, BTB/POZ and TAZ domain-containing protein. The lncRNA *MSTRS.107242.6* and its target gene *VaccDscaff4-snap-gene-377.47* were proposed to have a positive co-expressed relationship. Both transcripts showed lower expression levels in PAD and CUP fruits and higher expression levels in PINK fruits (**Figure 7**). Their expression was dramatically upregulated in BLUE fruits. The correlation coefficient between the expression levels of *MSTRG.107242.6* and anthocyanins content was 0.987, suggesting that *MSTRG.107242.6* may be related to anthocyanin accumulation during fruit development.

## Discussion

The blueberry fruit, a source of bioactive compounds, has positive effects on human health. Many efforts have been made to elucidate the molecular mechanism of bioactive chemical formation and accumulation during fruit development. Nevertheless, information on non-coding RNAs in blueberries is still largely limited. In the present study, we identified >20,000 lncRNAs in blueberry. Four developmental stages were selected to study DE-lncRNAs in blueberry fruits. The potential roles of the DE-lncRNAs in key pathways in fruit expansion and maturation were investigated. These identified lncRNAs improved blueberry genome annotation and will help in the study of non-coding RNAs in blueberry.

### Distinct characterization between long non-coding RNA and protein-coding RNA

In our study, blueberry lncRNAs were classified into four categories according to their genomic locations related to protein-coding genes, and the percentage of lincRNAs was the highest among all types of lncRNAs. The maximum proportion of lincRNAs was found in other species, but our results revealed that the proportion of lincRNAs was lower in blueberry (49.2% of lincRNAs) than that in tomato (81%) (Zhu et al., 2015), *C. melo* (85.7%) (Tian et al., 2019), and apple (65%) (Yang et al., 2019). Distinct characterizations between lncRNAs and mRNAs have been found in the majority of plant and animal species. We found that blueberry lncRNAs had shorter transcript lengths, shorter ORF sizes, fewer exons, and lower expression levels than protein-coding RNAs, which is consistent with previous studies in other fruit species (Zhang et al., 2017; Chen et al., 2021). Our research also found that blueberry lncRNAs have fewer isoforms, which indicates that alternative splicing (AS) of lncRNAs occurs at a lower frequency than that of mRNAs. The expression of lncRNAs in different samples was more stage specific than that of mRNAs, which is consistent with studies in strawberry (Kang and Liu, 2015) and locust (Li et al., 2020a).

We identified a greater number of DEGs than that of DE-lncRNAs during fruit development in blueberries. Because the expression levels of lncRNAs were lower than those of mRNA, some lncRNAs could not be detected by high-throughput sequencing. The distribution of DE-lncRNA numbers in the three comparison groups was similar to that of DEGs, and both displayed the highest numbers in CUP *vs*. PINK group. This implied that the transcript profiles of lncRNAs and mRNAs changed synchronously during biologically developmental phases.

### Putative target genes of differentially expressed long non-coding RNAs were related to fruit development

LncRNAs play comprehensive roles in shaping transcription during plant development or in response to environmental stimuli (Lucero et al., 2020). However, they function independently of their protein-coding potential and are distinct from the small RNA guides of RNA interference (RNAi) pathways (Wierzbicki et al., 2021). Because lncRNAs have a lower level of sequence conservation between species, it is difficult to interpret the putative function of lncRNAs through sequence information. However, we were able to look for cues in the proposed *cis*- and *trans*-target genes. In the present study, a large number of protein-coding genes were predicted to be lncRNA targets. Our analysis displayed that the number of *trans*-target genes was larger than that of *cis*- targets, and the KEGG annotation was distinct between two types of target genes.

The regulatory roles of lncRNA in fruit ripening have been demonstrated in previous studies. At the mature stage of sea buckthorn (Zhang et al., 2017), the target genes of DE-lncRNAs were enriched in carotenoid biosynthesis, ascorbate and aldarate metabolism, and fatty acid metabolism pathways. During fruit maturation and postharvest of kiwifruit (Chen et al., 2021), target genes of DE-lncRNAs were associated with the cell wall modification process, starch and sucrose metabolism, and plant hormone signal transduction. In our research, the KEGG enriched pathways in the three phases were coordinated to the morphological changes of the fruit. For example, during PAD to CUP phase, pathways related to “DNA replication” were enriched for *cis*-targets, implying that cells are undergoing robust division. The anthocyanin biosynthesis pathway was listed in KEGG enrichments of *cis*-targets during the PINK to BLUE stages, which related to fruit coloration. Photosynthesis and chlorophyll metabolism were the top pathways for the *trans*-targets of DE-lncRNAs. These photosynthesis-related genes were upregulated in green fruit and downregulated in pink and blue fruits, which were also associated with color change. In addition to anthocyanin biosynthesis, the degradation of chlorophylls is another major factor that contributes to fruit color turning in blueberry (Li et al., 2020b). Therefore, we made the interesting hypothesis that lncRNAs might be involved in the chlorophyll metabolism by interacting with proteins or other non-coding RNAs during fruit maturation.

### Co-localization analysis implying potential functions of long non-coding RNAs in fruit expansion and ripening

The potential regulatory roles of lncRNAs were further analyzed using co-localization networks. We found that *cis*-target genes related to fruit expansion and maturation are presented in the network. LncRNAs play a critical role in fruit development (Zhu et al., 2015; Li et al., 2018; Yang et al., 2019). In our study, from the early fruit expansion phase, genes related to zeatin synthesis had a higher expression level in CUP fruits and were then downregulated. This implied that lncRNAs may function in regulating zeatin accumulation and indirectly promoting cell division during fruit expansion stages. During the later fruit expansion stages (from CUP to PINK), we identified a larger number of regulatory lncRNAs that were related to cell expansion and color change by co-localization analysis. In addition to the top KEGG pathways of Plant-pathogen interaction and mitogen-activated protein kinase (MAPK) signaling pathway-plant, the pathways of Phenylalanine biosynthesis, Flavonoid biosynthesis, and Plant hormone signal transduction were presented in the CUP to PINK stages. The pathway of anthocyanin biosynthesis was enriched at the fruit ripening stage, which was related to the formation of a blue color. These results implied that lncRNAs may participate in flavonoid and anthocyanin biosynthesis during the fruit ripening stages.

### Potential function of long non-coding RNAs in flavonoid biosynthesis during fruit ripening

LncRNAs play regulatory roles in anthocyanin biosynthesis during fruit maturation in apple (Ma et al., 2021; Yu et al., 2022). In the present study, several lncRNAs were found to be *cis*-regulators of key genes involved in flavonoid biosynthesis. Zhang et al. (2017) also showed that DE-lncRNAs were enriched in flavonoid biosynthesis during fruit maturation of sea buckthorn. Our results revealed that *F3’5’H* was dramatically upregulated from PINK to BLUE and may be regulated by lncRNAs. *F3’5’H* is a class of cytochrome P450-dependent flavonoid hydroxylases that function in the hydroxylation of dihydroflavonol at the 3’ and 5’ positions of the B-ring. Previous studies (Guo et al., 2022) have shown that the *VcF3’5’H4* gene may play an important role in the light-induced blueberry anthocyanin synthesis pathway. Two *UFGT* genes that are involved in the conversion of anthocyanidins to anthocyanins were highly expressed in blue fruit but not in green fruit and were *cis*-targeted by lncRNAs with the same expression patten. These findings suggested that the expressions of *F3’5’H* and *UFGT* were upregulated to promote anthocyanin synthesis in blue fruits, which was supported by a previous study in blueberry (Zifkin et al., 2011). We discovered that the accumulation of flavonols was strongly correlated with the *flavonol synthase* gene *FLS* and its *cis*-regulatory lncRNA. Another study (Tang et al., 2021) also found that when blueberries ripened, their flavonol synthesis and *FLS* expression both dropped. The *LAR* gene, which plays a positive role in proanthocyanin accumulation, had a high expression level at the early stages. Still, its expression was not detected in the ripening stage, which is consistent with previous studies (Zifkin et al., 2011; Colle et al., 2019; Tang et al., 2021). When the expressions of *FLS* and *LAR* were inhibited during fruit ripening, the flavonol and proanthocyanidin syntheses were prevented, while anthocyanin biosynthesis was indirectly promoted. So, we speculated that *FLS* and *LAR* play negative roles in anthocyanin accumulation (**Figure 7**). However, the biological function of lncRNAs in the flavonoid synthesis pathway requires further experimental verification.

In our study, only one *cis*-regulated lncRNA (*MSTRG.107242.6*), which was presented in all three comparisons, might play important roles in fruit development. A high correlation coefficient between *MSTRG.107242.6* and anthocyanin content was found in the present study. The putative target gene of *MSTRG.107242.6* was proposed as a *BTB/POZ and TAZ domain-containing protein*. The TF BPM3 (BTB-POZ/MATH E3 ligase) mediates flavonoid biosynthesis in Tartary buckwheat (Ding et al., 2021). In apple, a member of the BTB/TAZ family (BT2) plays critical roles in the regulation of nitrate deficiency-induced anthocyanin accumulation (Ren et al., 2021). Therefore, BTB/POZ domain-containing proteins may have important functions in flavonoid biosynthesis and anthocyanin accumulation. The potential interaction between *MSTRG.107242.6* and *BTB/POZ and TAZ domain-containing protein* perhaps forms a posttranscriptional regulatory network underlying anthocyanin biosynthesis. These results suggest that lncRNAs may participate in complex networks underlying the posttranscriptional regulation of anthocyanin synthesis during blueberry fruit development.

## Conclusions

Using strand-specific RNA-seq, a total of 25,036 lncRNAs from 17,801 loci were identified during fruit development in the southern highbush blueberry cv. ‘Misty.’ Among these lncRNAs, 12,306 lincRNAs, 2,503 antisense lncRNAs, 6,372 intronic lncRNAs, and 3,855 sense lncRNAs were classified. Distinct characterizations between lncRNAs and protein-coding RNAs have been revealed in blueberry. Comparative analysis displayed that target genes of the DE-lncRNAs were related to cell proliferation, photosynthesis, chlorophyll metabolism, flavonoid biosynthesis, and anthocyanin synthesis during blueberry fruit development. During all stages, a large number of *trans*-targets were enriched for “photosynthesis” and “chlorophyll metabolism.” The top KEGG pathways that presented in the co-localization network involved “Zeatin biosynthesis” at the early fruit expansion stage, “Plant-pathogen interaction” and “MAPK signaling pathway” at the middle stage, and “Cysteine and methionine metabolism” and “Anthocyanin biosynthesis” at the ripening stage. Several lncRNAs are proposed as *cis-*regulators of the key genes involved in flavonoid and anthocyanin biosynthesis.

## Data availability statement

The datasets presented in this study can be found in online repositories. The names of the repository/repositories and accession number(s) can be found in the article/**Supplementary Material**. The Fastq files of the strand-specific transcriptome sequence for 12 samples have been deposited in National Center for Biotechnology Information (NCBI) BioProject database. This data can be found here: https://www.ncbi.nlm.nih.gov/bioproject/PRJNA846130.

## Author contributions

SL and JZ performed the experiments, analyzed the data and wrote the manuscript. XZ and HA organized the entire project. LZ, XF and JL revised and edited the manuscript. All authors contributed to the article and approved the submitted version.
