# Supplementary material for: Genome-wide identification and comprehensive analysis reveal potential roles of long non-coding RNAs in fruit development of southern highbush blueberry (Vaccinium corymbosum L.)
Source: Front Plant Sci. 2022 Dec 13;13:1078085. doi: 10.3389/fpls.2022.1078085 (PMC9792668; doi:10.3389/fpls.2022.1078085)
Supplement: Supplementary file 2 [file DataSheet_1.docx]

**Supplementary Table S1** The sequences of all primers used for qRT-PCR

| Transcripts | Primers (5’-3’) |
| --- | --- |
| GAPDH | AGAAAGAATACAAGCCAGAT  GGTAGTCATAAGACCCTCAA |
| MSTRG.29197.2 | ATGGATCTGCGTCTGCTCTG  CCTGAAAGCCAAGCCATTCG |
| MSTRG.80923.2 | GCAATACGCGCTCCAGTAAG  ACGCGACCAGTATAGCAACC |
| MSTRG.107242.6 | ACGACACCACGTGCTAACTG  ACAAACGGACTCCCAACAGG |
| MSTRG.42420.1 | TGAGGTGCTCGGAAATGGTC  GAACCAACCCCCTAAAGCGA |
| MSTRG.141621.4 | ACTTCCGGAGCTGCTTGAAT  GTCTGGGCTAAAGCCGCAAT |
| MSTRG.74005.4 | GTGTGCGTGTGTTCTTTGCT  CGCAATTTCGGACAGAACCG |
| VaccDscaff4-snap-gene-377.47 | CGTGTAGGCGAAGGGTGAAT  GCAAAGATTGGGCTGGTCAC |
| VaccDscaff30-augustus-gene-327.30 | ACGCGCTCCAGTAAGGTTTC  GCCGATCGCTTAGGTCATCC |
| VaccDscaff37-augustus -gene-300.34 | ATGGGCTCCTCAAGTGCAAG  CCACCTGATGATCCCCGAAG |

**Supplementary Table S2** Sequencing data quality statistics

| Samples | Clean reads | Q20 (%) | Q30 (%) | GC (%) | Mapped percentage (%) | Mapped to exon (%) | Mapped to intergenic (%) | Mapped to intron (%) |
| --- | --- | --- | --- | --- | --- | --- | --- | --- |
| PAD 1 | 75232004 | 98.11 | 94.78 | 43.46 | 75.47 | 60.5 | 24.5 | 15.0 |
| PAD 2 | 80959890 | 98.14 | 94.81 | 43.48 | 75.55 | 61.0 | 24.2 | 14.8 |
| PAD 3 | 67781082 | 98.31 | 95.13 | 43.01 | 69.44 | 56.2 | 25.8 | 18.0 |
| CUP 1 | 66559780 | 98.15 | 94.80 | 43.56 | 75.93 | 64.2 | 21.6 | 14.2 |
| CUP 2 | 64323960 | 98.33 | 95.26 | 44.01 | 76.70 | 65.9 | 20.2 | 13.8 |
| CUP 3 | 65050840 | 98.30 | 95.15 | 43.53 | 75.99 | 64.0 | 21.8 | 14.2 |
| GREEN 1 | 64814556 | 98.50 | 95.27 | 44.99 | 81.34 | 69.1 | 17.3 | 13.5 |
| GREEN 2 | 74420648 | 98.34 | 94.91 | 45.03 | 81.29 | 69.2 | 17.4 | 13.4 |
| GREEN 3 | 68396910 | 98.23 | 94.78 | 44.90 | 81.25 | 70.5 | 16.7 | 12.8 |
| BLUE 1 | 72755094 | 98.20 | 94.97 | 45.31 | 82.65 | 69.5 | 16.3 | 14.2 |
| BLUE 2 | 75457220 | 98.25 | 95.08 | 45.38 | 82.69 | 69.7 | 16.1 | 14.2 |
| BLUE 3 | 66826678 | 98.17 | 94.98 | 45.67 | 82.91 | 70.5 | 15.5 | 14.0 |
